# Correction: Resistive switching effect and magnetic properties of iron oxide nanoparticles embedded-polyvinyl alcohol film

**DOI:** 10.1039/d0ra90037k

**Published:** 2020-04-27

**Authors:** Hai Hung Nguyen, Hanh Kieu Thi Ta, Sungkyun Park, Thang Bach Phan, Ngoc Kim Pham

**Affiliations:** Faculty of Materials Science and Engineering, University of Science HoChiMinh City Vietnam phamkngoc@hcmus.edu.vn; Vietnam National University HoChiMinh City Vietnam; Center for Innovative Materials and Architectures HoChiMinh City Vietnam; Department of Physics, Pusan National University Busan Korea

## Abstract

Correction for ‘Resistive switching effect and magnetic properties of iron oxide nanoparticles embedded-polyvinyl alcohol film’ by Hai Hung Nguyen *et al.*, *RSC Adv.*, 2020, **10**, 12900–12907.

The authors regret that one of the affiliations (affiliation *a, b, c*) was incorrectly shown in the original manuscript. The corrected list of affiliations is as shown above.

The Royal Society of Chemistry apologises for these errors and any consequent inconvenience to authors and readers.

## Supplementary Material

